# PYK2 integrates growth factor and cytokine receptors signaling and potentiates breast cancer invasion via a positive feedback loop

**DOI:** 10.18632/oncotarget.4257

**Published:** 2015-06-10

**Authors:** Michael Selitrennik, Sima Lev

**Affiliations:** ^1^ Molecular Cell Biology Department, Weizmann Institute of Science, Rehovot, Israel

**Keywords:** breast carcinoma, invasion, signaling, IL-8, ErbB receptors

## Abstract

The involvement of ErbB family members in breast cancer progression and metastasis has been demonstrated by many studies. However, the downstream effectors that mediate their migratory and invasive responses have not been fully explored. In this study, we show that the non-receptor tyrosine kinase PYK2 is a key effector of EGFR and HER2 signaling in human breast carcinoma. We found that PYK2 is activated by both EGF and heregulin (HRG) in breast cancer cells, and positively regulates EGF/HRG-induced cell spreading, migration and invasion. PYK2 depletion markedly affects ERK1/2 and STAT3 phosphorylation in response to EGF/HRG as well as to IL8 treatment. Importantly, PYK2 depletion also reduced EGF/HRG-induced MMP9 and IL8 transcription, while IL8 inhibition abrogated EGF-induced MMP9 transcription and attenuated cell invasion. IL8, which is transcriptionally regulated by STAT3 and induces PYK2 activation, prolonged EGF-induced PYK2, STAT3 and ERK1/2 phosphorylation suggesting that IL8 acts through an autocrine loop to reinforce EGF-induced signals. Collectively our studies suggest that PYK2 is a common downstream effector of ErbB and IL8 receptors, and that PYK2 integrates their signaling pathways through a positive feedback loop to potentiate breast cancer invasion. Hence, PYK2 could be a potential therapeutic target for a subset of breast cancer patients.

## INTRODUCTION

Receptor tyrosine kinases (RTKs) of the ErbB family (ErbB1–4) are highly expressed in different breast cancer subtypes [1] and frequently contribute to breast cancer progression and metastasis [2]. Overexpression of ErbB2 (HER2), for example, is associated with a highly aggressive breast tumors and poor clinical outcome [3]. The ErbB receptors are activated by ligand-induced homo- or heterodimerization, followed by receptors trans-phosphorylation and activation of multiple downstream effectors and signaling cascades. Two classes of ligands activate the ErbB receptors; the EGF-like ligands and neuregulins (NRGs). EGF-like ligands induce the formation of ErbB1 (EGFR) homodimers or ErbB1/2 heterodimers, whereas NRGs bind directly to ErbB3 and/or ErbB4 and induce the formation of ErbB3 or ErbB4 homodimers as well as ErbB2/3 or ErbB2/4 heterodimers [4]. ErbB2 does not bind any known ligand [5], and ErbB3 has a crippled kinase domain lacking catalytic activity [6], and thus these two receptors actively function as heterodimers. In fact, ErbB2, which dimerizes with other ErbB receptors, functions as a signal amplifier by inhibiting the rate of growth factor dissociation as well as of receptors endocytosis and lysosomal degradation, and by enhancing the recycling of internalized receptors back to the cell surface [7, 8]. ErbB2/3 heterodimer is known to induce strong mitogenic response [9], and ErbB2 in general, plays a critical role in heregulin (HRG)-induced breast cancer cell growth and migration [10]. Recent studies suggest that ErbB2 as well as EGFR can also be trans-activated by cytokines including Interleukin-8 (IL8; CXCL8) [11], a neutrophil chemoattractant and an angiogenic factor with tumor promoting properties [12, 13].

IL8 is secreted by cells of the tumor microenvironment, including endothelial cells, infiltrating neutrophils, and tumor-associated macrophages, or by the tumor cells themselves. IL8 binds to the G protein-coupled receptors (GPCR) CXCR1 and CXCR2 [13], and activates multiple signal transduction cascades that can affect cell survival, migration and invasion. Previous studies have shown that IL8 induces activation of the non-receptor tyrosine kinase PYK2 in human neutrophils and that PYK2 activation is crucial for IL8-induced neutrophil chemotaxis [14].

PYK2 is activated by multiple cytokines, growth factors (GFs), hormones and neuropeptides [15–18] and regulates different signal transduction cascades that control cell proliferation, migration and invasion [19–21]. PYK2 has been implicated in the progression and invasion of several human cancers, including glioblastoma, hepatocytoma, non-small-cell lung carcinoma, prostate as well as breast cancer [22, 23]. Moreover, previous studies showed that PYK2 and its closely related kinase FAK are highly expressed in ErbB2-positive breast cancers and contribute to the proliferative and invasive potential of breast cancer cell lines [24, 25]. We have recently found that PYK2 enhances epithelial-to-mesenchymal transition (EMT) and markedly facilitates the migration and invasion of triple-negative breast cancer (TNBC) cells [26]. Specifically we found that PYK2 prolongs the signals of EMT-inducers, such as EGF, by sustaining endosomal-derived receptor signaling and consequently postponing signal termination, and by participating in a positive feedback loop that links cell surface receptor(s) to transcription factor(s) activation. This positive feedback loop links the EGFR and cMet RTKs to PYK2 activation, which in turn phosphorylates STAT3 who directly regulates PYK2 transcription [26]. The involvement of PYK2 in a positive feedback loop suggests that PYK2 can integrate different signal transduction cascades and potentiate various cellular responses. Indeed, we have previously showed that PYK2 functions at a convergence point between integrin and GPCRs signaling [27]. Here we show that PYK2 integrates signaling of the ErbB receptors and the IL8 CXCR1/2 receptors to potentiate breast cancer cell invasion through a positive feedback loop.

## RESULTS

### EGF and HRG induce activation of PYK2 in different breast cancer cell lines

We have recently showed that EGF induces rapid phosphorylation of PYK2 in the TNBC cell line MDA-MB-468, and that PYK2 positively regulates EGF-induced migration and invasion of MDA-MB-468 cells [26]. To examine the role of PYK2 in ErbB receptors signaling in other breast cancer subtypes, we employed two estrogen receptor (ER) positive luminal A breast cancer cell lines, MCF7 and T47D, and an HER2-overexpressing cell line, SKBR3. MCF7 cells express low levels of EGFR and HER2, while T47D express moderate levels of the four ErbB receptors (EGFR/ErbB1, ErbB2/HER2, ErbB3/HER3, and ErbB4/HER4 receptors) [28, 29]. SKBR3 cells overexpress HER2, highly express EGFR but have relatively low levels of ErbB3 and ErbB4 [30] (Supplementary Figure 1).

**Figure 1:**
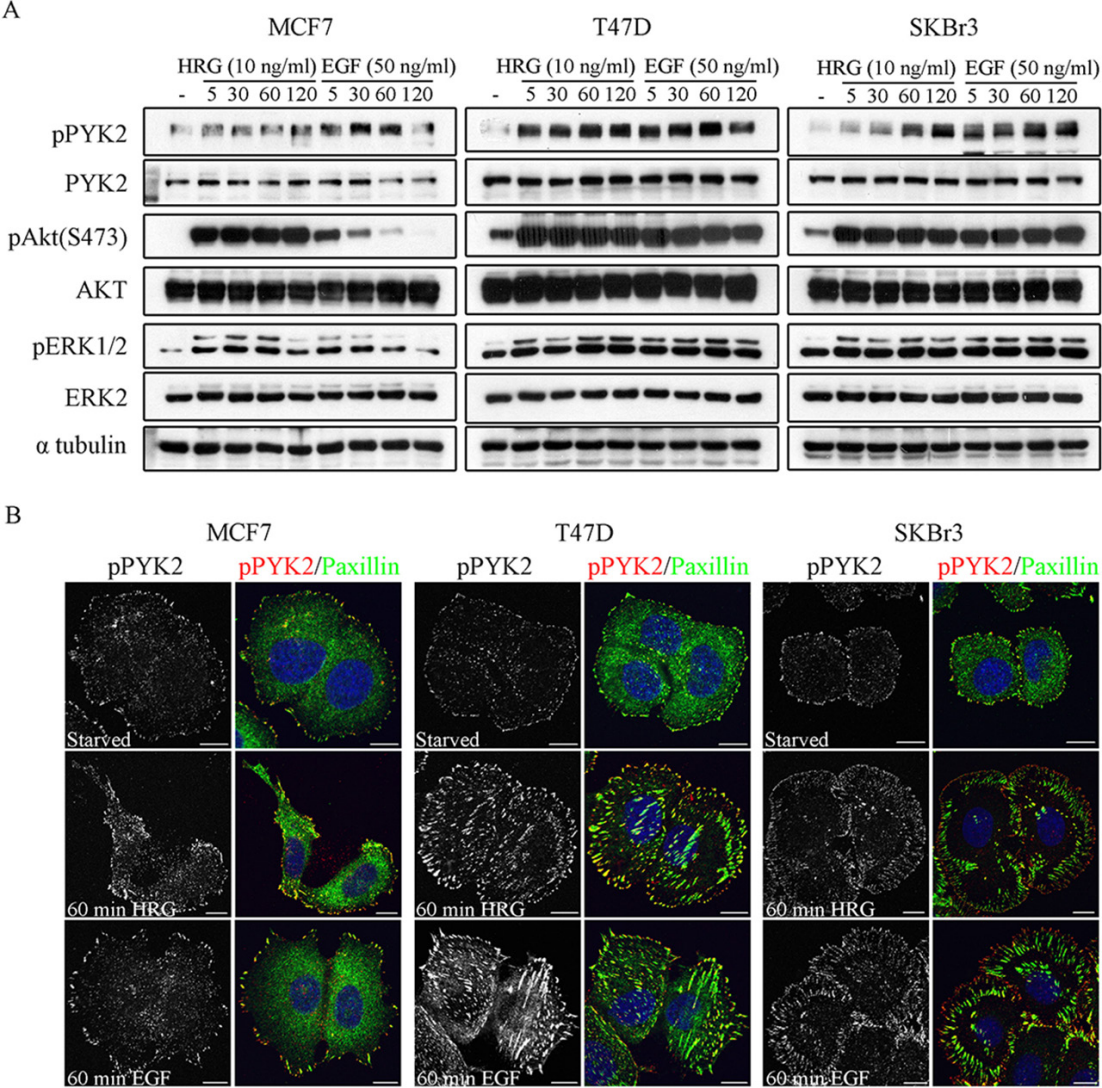
Phosphorylation and subcellular localization of PYK2 in EGF- and HRG-stimulated breast cancer cells **A.** Phosphorylation of PYK2. MCF7, T47D and SKBR3 cells were serum-starved for 24 hr and then stimulated with EGF (50 ng/ml) or HRG (10 ng/ml) for the indicated time periods. Total cell lysates were prepared and analyzed for PYK2 phosphorylation (pY402) as well as phosphorylation of ERK1/2 and AKT (S473) by WB using phospho-specific antibodies. Reproducible results were obtained in three independent experiments. Quantitative analysis of these results is shown in Supplementary Figure 2. **B.** Localization of pPYK2 (pY402). MCF7, T47D and SKBR3 cells were serum-starved for 24 hr and then stimulated with the indicated growth factors for 5–60 min. The localization of phospho-PYK2 was assessed by immunostaining with anti-phospho-PYK2 (Y402) antibody. Staining for paxillin was used as a focal adhesion marker. Shown are representative confocal images. Scale bars: 10 μm.

Previous studies have shown that EGF and HRG enhance the migration of these three breast cancer cell lines [29, 31, 32], as well as the invasion of MCF7 and SKBR3 cells [32]. To define the role of PYK2 in ErbB receptors-mediated signaling, we first examined whether EGF and HRG induce phosphorylation of PYK2 at its major autophosphorylation site Y402 utilizing a phospho-specific antibody and Western blotting (WB) analysis. As shown in Figure 1A and in Supplementary Figure 2, both EGF and HRG induced rapid phosphorylation of PYK2 in the three breast cancer cell lines. Phospho-PYK2 (pY402) was already detected at 5 min following ligand stimulation and sustained for almost 2 hr. Importantly, EGF and HRG also induced phosphorylation of ERK1/2 in the three cell lines as assessed by WB using anti-pERK1/2 antibody. Likewise, HRG induced AKT phosphorylation (S473) in the three cell lines, while EGF induced strong and sustained phosphorylation of AKT in SKBR3 and T47D cells (2 hr) and transient AKT phosphorylation in MCF7 cells. These results suggest that both EGF and HRG induce PYK2 phosphorylation and concomitantly activate the MAPK and the PI3K/AKT pathways, two signaling pathways that regulate the migration and/or invasion of these breast cancer cells [29, 32].

We next examined the subcellular distribution of phospho-PYK2 in serum-starved or EGF/HRG-stimulated cells using immunofluorescence (IF) and confocal microscopy analysis. Representative images of control and ligand-induced cells at 60 min are shown in Figure 1B. As seen, both EGF and HRG markedly increased the focal adhesion sites as well as the phosphorylation (pY402) of PYK2 as determined by immunostaining for paxillin and PYK2(pY402), respectively. Interestingly, pPYK2 was mainly detected in peripheral focal adhesions and partially co-localized with paxillin, suggesting that these two proteins co-localized at subsets of focal adhesions, and also that they are both involved in cell migration.

### PYK2 positively regulates EGF/HRG-induced breast cancer cell spreading and migration

To examine the effect of PYK2 on EGF- or HRG-induced migration of SKBR3, T47D or MCF7 cells, we knocked down its expression by shRNA using lentivirus infection as we previously described [26]. Two PYK2 shRNAs (Supplementary Figure 3) have been used to downregulate the expression of PYK2 throughout this study, and representative results with only one shRNA are shown.

**Figure 2:**
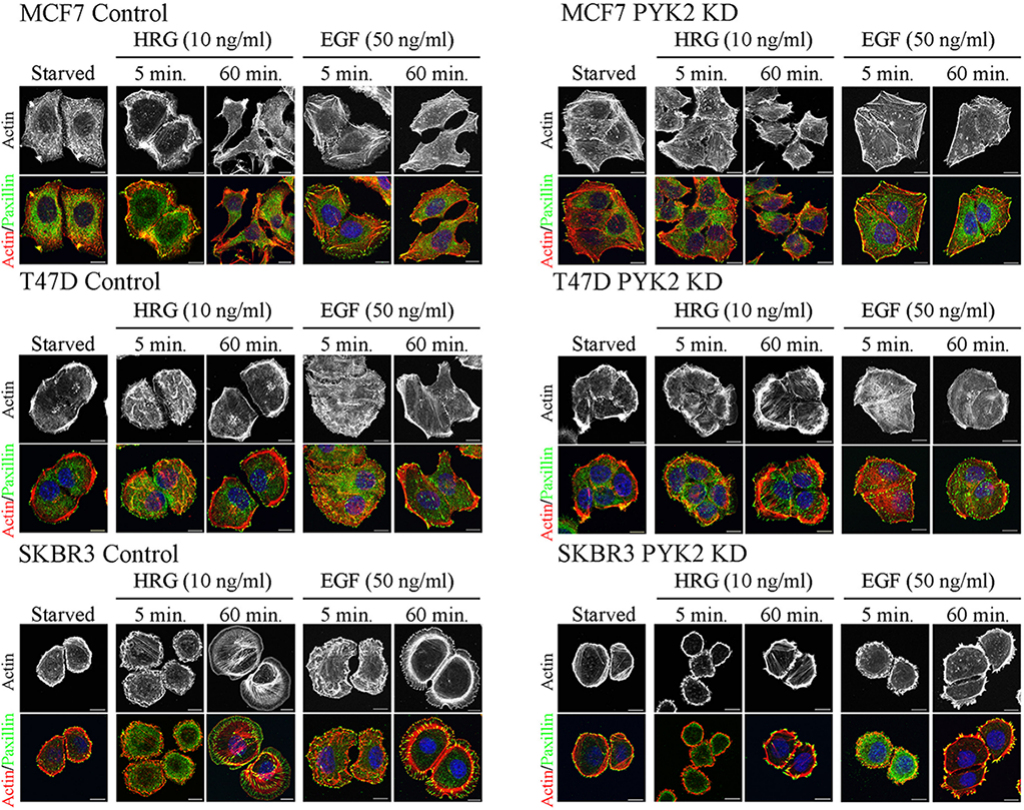
PYK2 depletion affects EGF/HRG-induced breast cancer cell spreading and actin cytoskeleton remodeling Control and PYK2-depleted MCF7, T47D and SKBR3 cells were serum-starved for 24 hr and then stimulated with the indicated growth factors for 5–120 min. The cells were then fixed and double stained with TRITC-phalloidin (red) as a F-actin marker and anti-paxillin (green) antibody as a focal adhesion marker. Shown are representative confocal images. Scale bars: 10 μm.

Cell migration is a multistep process involving a series of morphogenetic events and actin cytoskeleton remodeling [33]. We therefore examined the influence of PYK2 knockdown on EGF- or HRG-induced cell spreading and actin cytoskeleton remodeling using IF and confocal microscopy analysis. As shown in Figure 2, both HRG and EGF induced rapid (5 min) cells spreading and membrane ruffling in the control MCF7, T47D and SKBR3 cells. Later the cells extended lamellipodia together with the formation of actin stress fibers (60 min), consistent with previous reports [29]. However, PYK2-depleted MCF7, T47D, or SKBR3 cells failed to spread in response to either EGF or HRG and remained tightly in contact even 60 min following ligand stimulation. Membrane ruffles and lamellipodia could hardly be detected in either EGF- or HER-induced PYK2-depleted cells, suggesting that PYK2 depletion markedly affects early events of the migratory response in these three breast cancer cell types.

**Figure 3:**
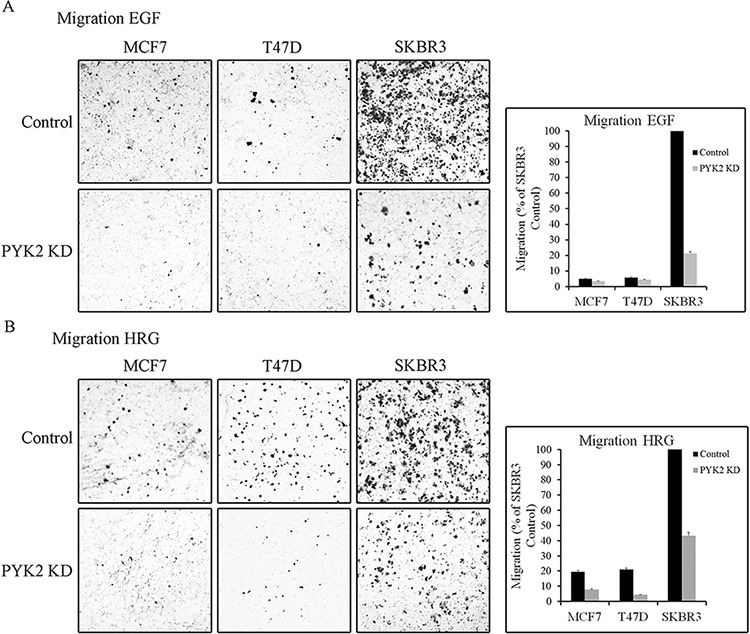
PYK2 depletion inhibits EGF/HRG-induced breast cancer cell migration Migration of control and PYK2-depleted MCF7, T47D and SKBR3 cells in response to EGF (50 ng/ml) **A.** or HRG (10 ng/ml) **B.** was assessed by Boyden chamber assay. Representative images of the migrated cells are shown along with plotted graphs demonstrating the mean values ± s.d. from three independent experiments.

We next examined whether PYK2 affects the migration of MCF7, T47D or SKBR3 cells in response to either EGF or HRG employing the transwell migration assay. As seen in Figure 3, the migratory response to either EGF or HRG was most profound in the control SKBR3 cells. HRG had a stronger effect on the migration of T47D and MCF7 cells as compared to EGF, consistent with the low expression levels of EGFR in these cells (Supplementary Figure 1). Depletion of PYK2 markedly attenuated the migratory responses to either EGF or to HRG in the three cell lines. These findings suggest that PYK2 not only regulates early migratory events, such as cell spreading and membrane ruffling, but also has a profound effect on EGF- and/or HRG-induced breast cancer cell migration.

### PYK2 affects EGF- and HRG-mediated downstream signaling in breast cancer cells

**Figure 4:**
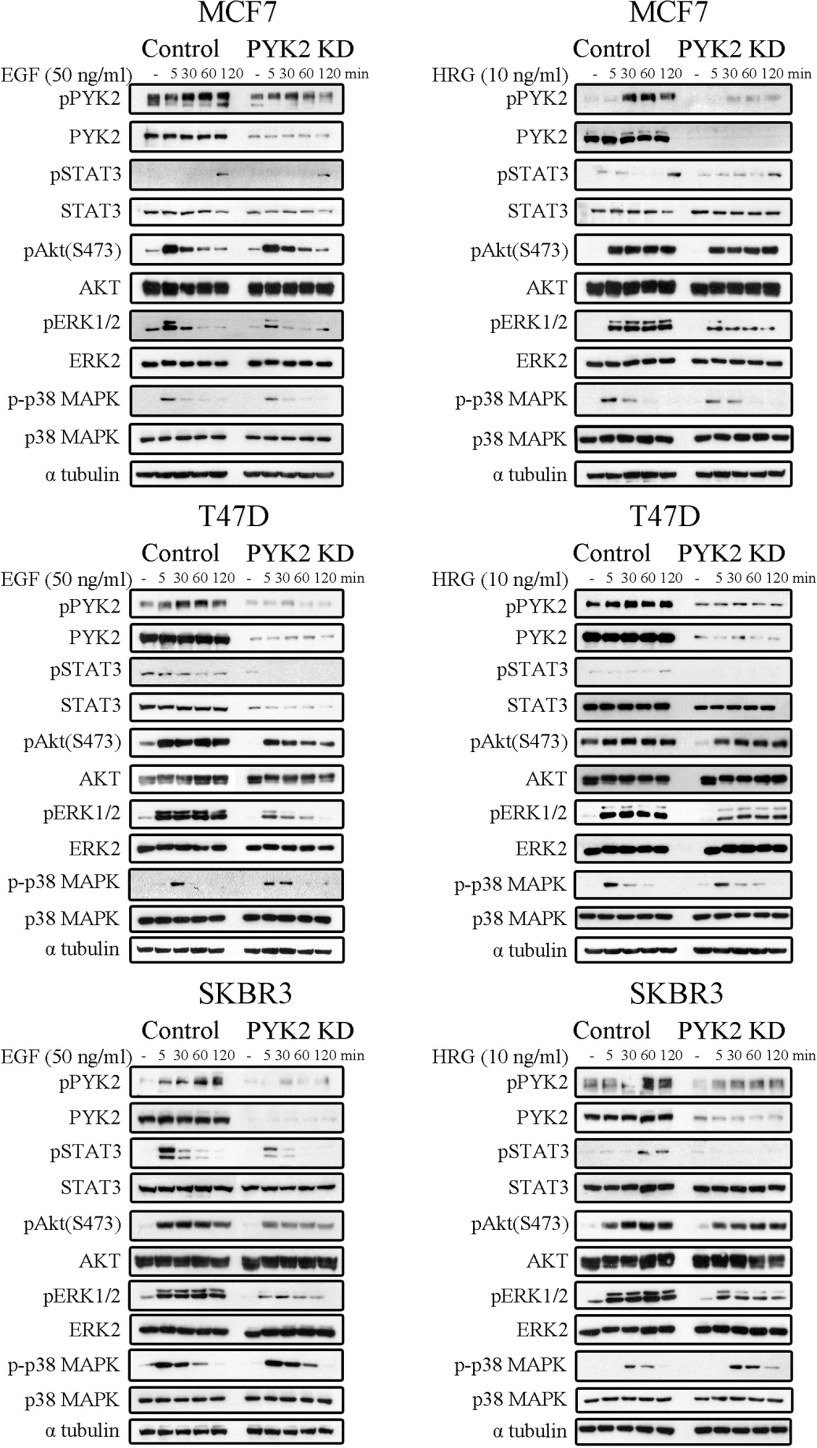
PYK2 depletion inhibits EGF/HRG-downstream signaling in breast cancer cells Control and PYK2-depleted MCF7, T47D and SKBR3 cells were serum-starved for 24 hr and then stimulated with EGF (50 ng/ml) or HRG (10 ng/ml) for the indicated time periods. Total cell lysates were prepared and analyzed for the activation of different signaling pathways using WB analysis and phospho-specific antibodies as indicated. Quantitation of these results is shown in Supplementary Figure 4. Reproducible results were obtained in three independent experiments.

Previously it was shown that multiple signaling pathways regulate the migration and/or invasion of MCF7 [34], T47D [29], and SKBR3 [32] cells in response to HRG and EGF. The Ras/MAPK, PI3K/AKT, p38MAPK and the STAT3 pathways have been implicated in the migratory and invasive responses of EGF and/or HRG in these cell lines [29, 35, 36]. To examine the influence of PYK2 on these signaling pathways, we compared the phosphorylation of ERK1/2, p38MAPK, AKT (S473) and STAT3 (Y705) in control and PYK2-depleted cells in response to EGF or HRG using phospho-specific antibodies. As seen in Figure 4 and in Supplementary Figure 4, knockdown of PYK2 markedly reduced (by 70–90%, Supplementary Figure 4) EGF- or HRG-induced ERK1/2 phosphorylation in all the three cell lines. AKT phosphorylation (S473) was slightly affected by PYK2 knockdown in T47D and SKBR3 cells in response to EGF (by 40% and 30%, respectively, Supplementary Figure 4) but not in response to HRG. STAT3 phosphorylation, which was rapidly induced in EGF-treated SKBR3 cells, was also inhibited by PYK2 knockdown. STAT3 phosphorylation was weakly detected in the non-invasive T47D cells or the weakly invasive MCF7 cells in response to either EGF or HRG treatment, and PYK2 knockdown had a slight effect on EGF-induced STAT3 phosphorylation in T47D cells. Phosphorylation of p38 MAPK pathway was not inhibited by PYK2 knockdown. Collectively, these results suggest that PYK2 affects different migratory and invasive pathways in breast cancer cells in response to either EGF or HRG stimulation, but most profoundly affects the MAPK and STAT3 pathways.

### PYK2 depletion affects cell invasion and MMPs expression

**Figure 5:**
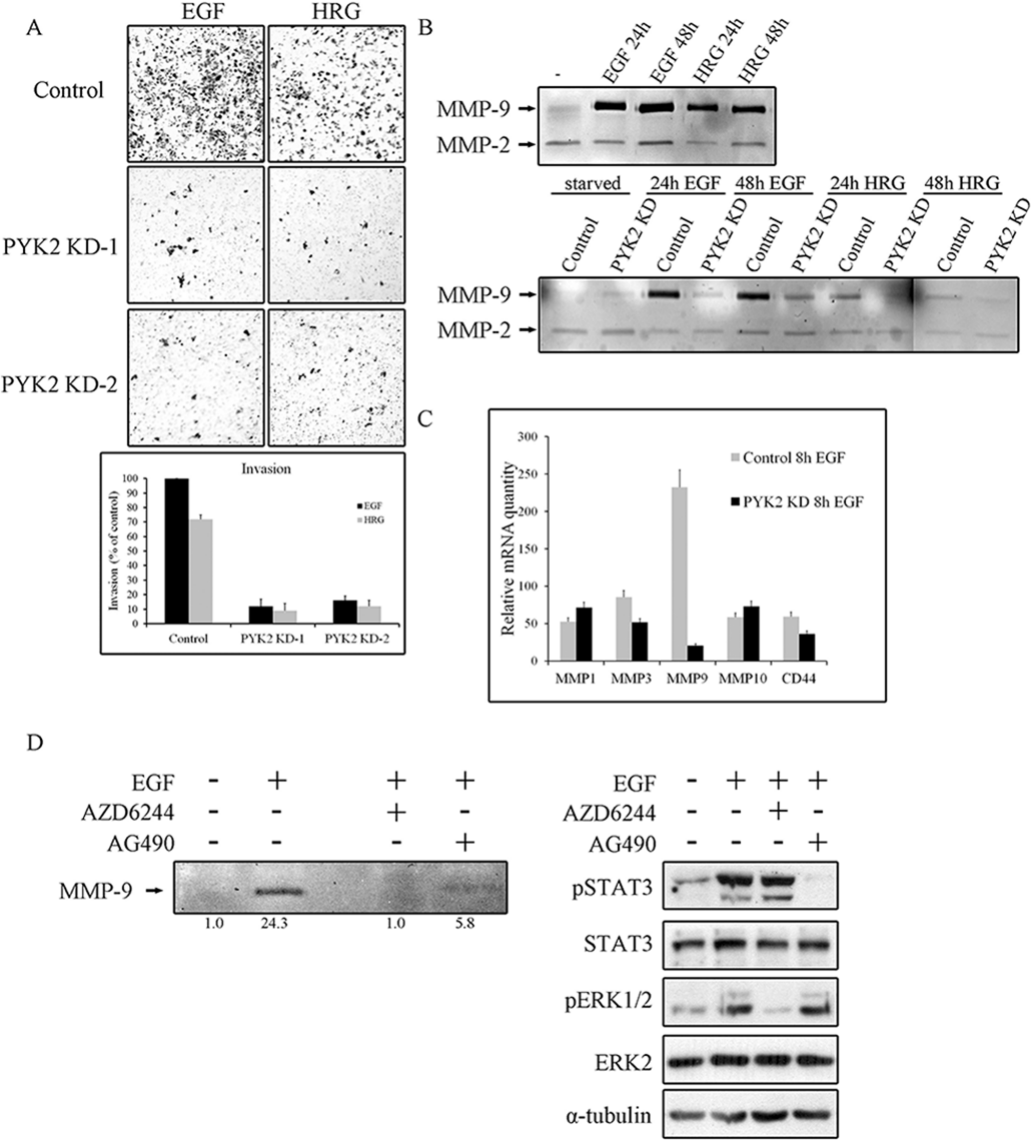
PYK2 depletion inhibits cell invasion and MMPs expression **A.** Invasion of control and PYK2-depleted SKBR3 cells through matrigel in response to EGF (50 ng/ml for 48 hr) or HRG (10 ng/ml for 48 hr) was assessed by the Boyden chamber assay. Representative images of the invasive cells are shown along with plotted graph demonstrating the mean values ± s.d. from three independent experiments. **B.** The activity of MMP2 and MMP9 in control and PYK2-depleted SKBR3 cells in response to EGF (50 ng/ml) or HRG (10 ng/ml) was examined by gelatin zymography assay. The activity was assessed in the conditioned cell medium after 24 and 48 hrs of incubation in serum-free medium. **C.** The mRNA levels of the indicated MMPs and MMP-related genes in the control and PYK2-depleted SKBR3 cells in response to EGF (50 ng/ml) were assessed by real-time PCR. Reproducible results were obtained in three independent experiments. The mRNA levels are relative to non-stimulated control cells. The mean values ± s.d. of three experiments are shown. **D.** The activity of MMP9 in EGF (50 ng/ml)-stimulated SKBR3 cells in the absence or presence of either the MEK inhibitor AZD6244 (5 μM) or JAK2/STAT3 inhibitor AG490 (10 μM) was examined by gelatin zymography as described in B. Densitometric analysis of the zymography bands was determined using NIH ImageJ software. The specificity of AZD6244 and AG490 inhibitors was assessed by WB analysis using phospho-specific antibodies as indicated.

It was previously shown that both EGF and HRG enhance the invasion of the highly metastatic SKBR3 cells [37]. Since PYK2 affects EGF/HRG-induced downstream signaling in SKBR3 cells (Figure 4), we examined the effect of PYK2 knockdown on SKBR3 invasion using the transwell invasion assay. As shown in Figure 5A, HRG elicited a weaker (∼70%) invasive response in SKBR3 cells as compared to EGF, and PYK2 depletion markedly (∼90%) attenuated SKBR3 invasion in response to either EGF or HRG treatment.

Invasion of cancer cells through basement membrane, tumor stroma, and blood vessel walls, requires the degradation of the extracellular matrix (ECM) by matrix metalloproteinases (MMPs) [38]. Previous studies have shown that EGF and HRG upregulate MMP9 in SKBR3 cells [39] as well as in MCF7 cells [40]. In contrast, the activity of the closely related MMP2 was unaffected by either HRG or EGF treatment [41]. By utilizing zymography assay we found that EGF strongly enhanced MMP9 activity in SKBR3 cells and only weakly activated MMP2 (Figure 5B), consistent with previous reports. We further found a weaker induction of MMP9 in response to HRG in SKBR3 cells (Figure 5B) and in response to EGF in MCF7 cells (Supplementary Figure 5). Most importantly, knockdown of PYK2 completely abolished EGF- or HRG-induced MMP9 activity (Figure 5B), suggesting that PYK2 attenuates cell invasion by inhibiting MMP9 activity. To examine whether PYK2 affects the activity of MMP9 or its transcription level, we first assessed the mRNA level of MMP9 in SKBR3 cells at different time points following EGF treatment using real-time PCR (qRT-PCR), and found a peak of induction at 8 hr (Supplementary Figure 6A). We then examined the influence of EGF on MMP9 transcription as well as on the transcription of additional MMPs (MMP-1, MMP-2, MMP-3, MMP-7, MMP-10, MMP-13, MMP14, MMP15) and of CD44, a cell adhesion glycoprotein implicated in EMT and cancer metastasis [42]. We observed that EGF markedly increased the transcription of MMP1, MMP3, MMP10, MMP9 and CD44 (by ∼50–250 fold) in SKBR3 cells. EGF had most profound effect on MMP9 transcription (∼250 fold), and most importantly, knockdown of PYK2 almost completely abolished this inducible effect of EGF on MMP9 transcription (Figure 5C), suggesting that PYK2 positively regulates MMP9 transcription and consequently affects its activity in EGF/HRG-induced SKBR3 cells.

### PYK2 potentiates cell invasion via a positive feedback loop between EGF and IL8 receptors

Thus far our findings suggest that PYK2 positively regulates the invasion of SKBR3 cells in response to EGF and HRG, at least in part, by increasing MMP9 transcription. Previous studies suggest that MMP9 transcription is regulated by multiple pathways, including the MAPK [37] and the STAT3 [43] signaling pathways. The involvement of STAT3 in EGF-induced MMP9 transcription in SKBR3 is controversial [44]. However, we found that inhibition of STAT3 by the JAK2/STAT3 inhibitor AG490 markedly reduced (∼3-fold) MMP9 activity as determined by zymography assay (Figure 5D). Yet, the MEK inhibitor AZD6244 had a more profound effect (Figure 5D), suggesting that the STAT3 pathway and more profoundly the MAPK pathway positively regulate MMP9 transcription in EGF-induced SKBR3 cells. The specificity and potency of both inhibitors (AG49 and AZD6244) were confirmed by Western blotting as shown in Figure 5D (lower panel).

**Figure 6:**
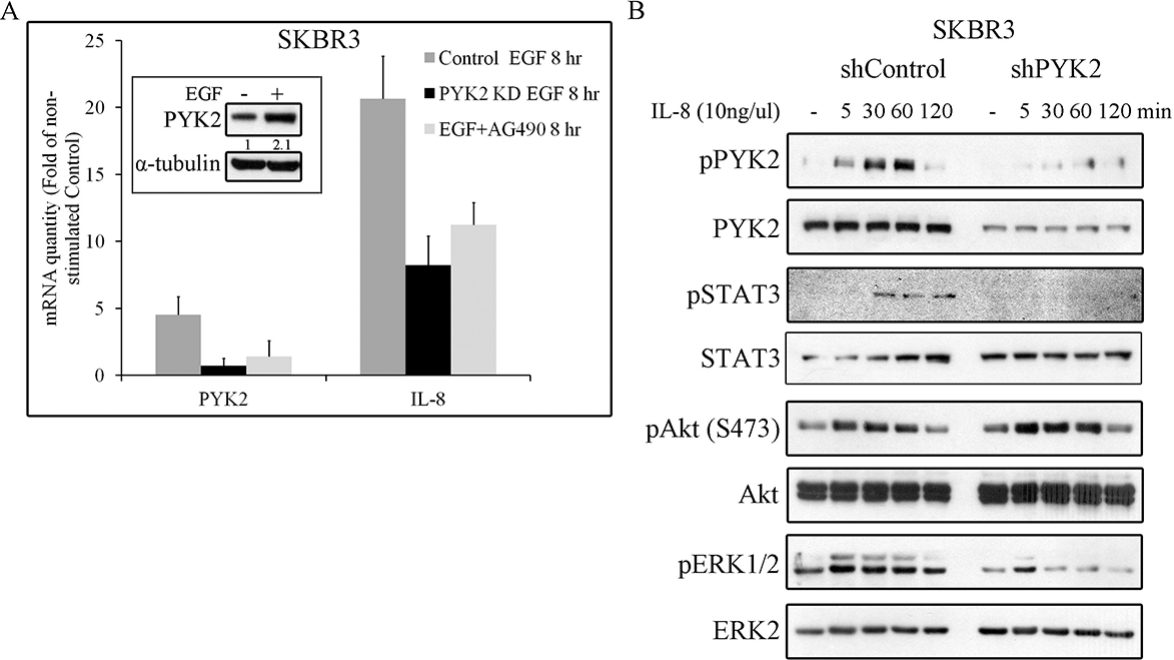
PYK2 affects EGF-induced IL8 expression while IL8 induces PYK2 phosphorylation **A.** EGF-induced IL8 expression in STAT3 and PYK2-dependent manner. The mRNA levels of PYK2 and IL8 in the control, AG490-treated (10 μM) and PYK2-depleted SKBR3 cells were assessed in response to EGF (50 ng/ml, 8 hr) stimulation using real-time PCR. The mRNA levels are presented as fold of un-stimulated control cells. The mean values ± s.d. of three experiments are shown. An insert of PYK2 protein levels in control serum-starved and EGF-stimulated cells is shown. Quantification was done as in Figure 5D **B.** IL8 induces phosphorylation of PYK2 and activates downstream pathways. Control and PYK2-depleted SKBR3 cells were serum-starved for 24 hr and then stimulated with IL8 (10 ng/ml) for the indicated time periods. Total cell lysates were prepared and analyzed for the activation of different signaling pathways using WB analysis and phospho-specific antibodies as indicated. Reproducible results were obtained in three independent experiments.

STAT3 is a transcription factor that regulates the expression of multiple targets genes including PYK2 [26] and the cytokine IL8. Previous studies suggest that IL8 plays a key role in HER2-mediated cell invasion [45]. We therefore assessed the effect of EGF on the transcription levels of both PYK2 and IL8 in SKBR3 cells. We found a remarkable upregulation of IL8 (∼17 fold) transcription and a moderate increase in the level of PYK2 mRNA (by 4 fold) and protein (Figure 6A). Inhibition of STAT3 activation by the JAK2/STAT3 inhibitor AG490 markedly decreased the upregulation of PYK2 and IL8 transcription in response to EGF, suggesting that STAT3 controls their mRNA expression. Likewise, depletion of PYK2 markedly reduced the effect of EGF (by ∼60%) on IL8 transcription, suggesting that PYK2 not only modulates MMP9 transcription in response to EGF but also positively regulates EGF-induced IL8 transcription. Furthermore, these observations suggest that EGF induces an IL8 autocrine signaling that may potentiate EGF-mediated responses. To explore this possibility, we first examined the effect of IL8 on different signaling pathways in SKBR3 cells including PYK2 phosphorylation. As shown in Figure 6B, IL8 induced a rapid phosphorylation of PYK2 (pY402) as well as phosphorylation of AKT (S473), ERK1/2 and STAT3 (Y705). Importantly, depletion of PYK2 substantially reduced the phosphorylation of ERK1/2 and STAT3 but had no effect on AKT phosphorylation. These results suggest that PYK2 is activated not only by EGF but also by EGF-induced IL8 secretion.

**Figure 7:**
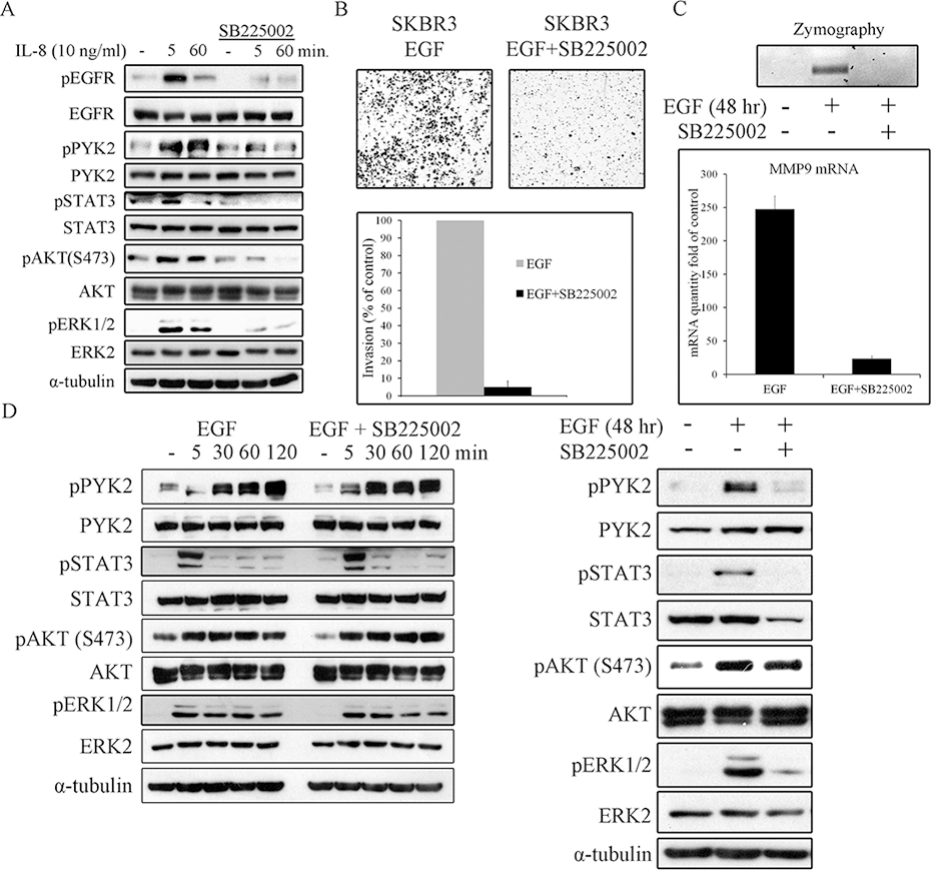
IL8 is essential for EGF-induced cell invasion, for MMP9 expression, and for prolonging downstream signaling SKBR3 cells were serum-starved for 24 hr and then stimulated with IL8 (10 ng/ml) **A.** or EGF (50 ng/ml) **D.** in the absence or presence of the CXCR2 inhibitor SB225002 (2 μM) for the indicated time periods. Total cell lysates were prepared and analyzed for the activation of different signaling pathways by WB using the indicated antibodies. Reproducible results were obtained in three independent experiments. **B.** Invasion of EGF (50 ng/ml)-stimulated SKBR3 cells through matrigel was assessed by the Boyden chamber assay in the absence or presence of SB225002 (2 μM). Representative images of the invasive cells are shown along with plotted graph demonstrating the mean values ± s.d. from three independent experiments. **C.** The activity and mRNA level of MMP9 in EGF (50 ng/ml)-stimulated SKBR3 cells in the absence or presence of SB225002 (2 μM) was examined by gelatin zymography and by real-time PCR assays, respectively. The activity and mRNA level were assessed in the conditioned cell medium after 48 hr of incubation in serum-free medium.

We next asked whether EGF-induced IL8 secretion plays a role in EGF-mediated cell invasion. To this end, we employed the SB225002 inhibitor, a competitive inhibitor of the IL8 receptor CXCR2, as CXCR2 is highly expressed SKBR3 cells [46]. This inhibitor has been previously used to block IL8-mediated signaling pathways [47, 48]. To demonstrate its potency and specificity, we examined its influence on IL8-induced PYK2, STAT3, AKT and ERK1/2 phosphorylation, and found that these IL8-downstream signals were markedly inhibited (Figure 7A). Importantly, IL8 also induced EGFR phosphorylation, which was substantially inhibited by SB225002, thus demonstrating the trans-activation of EGFR by CXCR1/2-mediated signaling, consistent with previous reports [11, 49].

Next, we examined the effect SB225002 on EGF-induced invasion of SKBR3 cells using the transwell invasion assay. As shown in Figure 7B, SB225002 markedly attenuated the invasion of SKBR3 cells in response to EGF. Importantly, SB225002 also inhibited EGF-induced MMP9 activation and its transcription upregulation as demonstrated by zymography assay (Figure 7C, upper panel) and by qRT-PCR (Figure 7C, lower panel), respectively. The marked effect of both PYK2 shRNA (Figure 5) and SB225002 on EGF-induced SKBR invasion and MMP9 expression, led us to examine the influence of SB225002 on EGF-induced phosphorylation of PYK2 as well as the phosphorylation of STAT3, AKT and ERK1/2. SKBR3 cells were treated with EGF in the absence or presence of SB225002 for either short time periods (5–120 min) or for 48 hr, typical time length of our invasion assay. The phosphorylation of PYK2, STAT3, AKT and ERK1/2 was assessed by WB using phospho-specific antibodies. As shown in Figure 7D (left panel), SB225002 had no obvious effects on EGF-mediated signals at early time points (5–120 min), as expected since the peak of IL8 expression was observed 8 hr following EGF treatment (Supplementary Figure 6B). However, at 48 hr, SB225002 significantly inhibited the phosphorylation of PYK2, STAT3 and ERK1/2 in response to EGF treatment, but had no effect on EGF-induced AKT phosphorylation (Figure 7D, right panel). These results imply that following EGF stimulation, PYK2 enhances a STAT3-dependent IL8 expression, thus creating a positive feedback loop between ErbB receptors, PYK2, and IL8. We propose that this positive feedback loop potentiates EGF-induced cell invasion.

## DISCUSSION

In this study we show that PYK2 integrates EGFR/HER2- and IL8-receptor signaling to potentiate cell invasion in breast cancer cells. Specifically, we found that PYK2 is activated in response to EGF and HRG in different breast cancer subtypes (Figure 1) and that PYK2 depletion leads to substantial inhibition of EGF/HRG-mediated cell spreading, migration and invasion (Figures 2, 3 and 5A). Although previous studies suggest that PYK2 is involved in the migration and invasion of glioma and breast cancer cells [21, 50, 51], the underlying mechanisms have not been fully explored. Here we show that PYK2 exerts its effects on breast cancer migration and invasion predominantly through the STAT3 and MAPK signaling pathways (Figure 4). Both pathways have been implicated in the transcriptional regulation of MMP9 in SKBR3 cells [37, 43]. Indeed, we found the MMP9 activity was markedly reduced by the MEK inhibitor and to a lesser extent by the Jak2/STAT3 inhibitor (Figure 5D).

MMP9 is an important mediator of tumor invasion and angiogenesis, and is significantly associated with high breast cancer metastasis and relapse [52]. Consistent with this, we found that both EGF and HRG induce a remarkable upregulation of MMP9 transcription as well as its activity in the highly metastatic SKBR3 cells, while exerting a minor effect on MMP9 expression in the poorly- and non-invasive MCF7 and T47D cells (Supplementary Figure 7). Most importantly, we found that PYK2 depletion markedly reduced EGF/HRG-induced MMP9 transcription and its subsequent gelatinase activity in SKBR3 cells, as determined by zymography assay (Figure 5B), implying that PYK2 plays an important role in breast cancer metastasis. Indeed, recent studies have shown that knocking down of PYK2 inhibits lung metastasis of breast cancer cells in mice xenograft model [53], and our analysis of human breast cancer tissues revealed that PYK2 expression correlates with high tumor grade and lymph node metastasis [26]. Furthermore, we found that PYK2 enhances EMT of breast cancer cells, a process that is associated with enhanced cell motility, invasiveness and metastatic propensity [26].

Various MMPs, inflammatory cytokines, chemokines and angiogenic factors, such as IL6, IL8, CXCL1, RANTES, and VEGF are secreted from tumor cells during EMT [54]. These secreted factors play important role in EMT maintenance and metastatic response. IL8, for example, is secreted in response to EMT inducers such as TGFβ or SNAIL overexpression and is involved in EMT maintenance through a positive autocrine loop [55]. We found that both EGF and HRG induce upregulation of IL8 transcription in SKBR3 cells but not in MCF7 or T47D cells (Supplementary Figure 7). Strikingly, we also found that IL8 is involved in a positive autocrine loop in SKBR3 cells.

**Figure 8:**
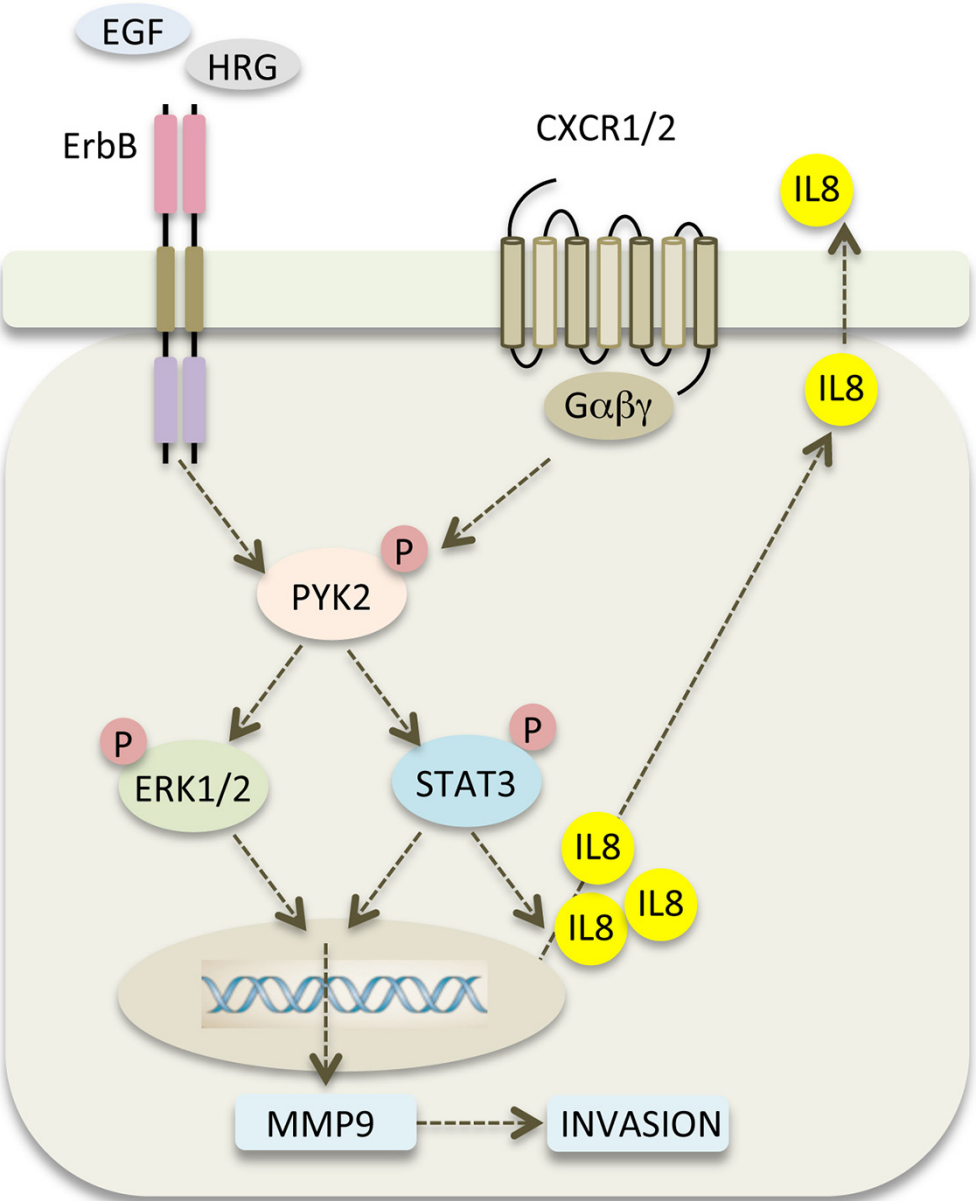
PYK2 links ErbB and IL8 receptors signaling to potentiate cell invasion A scheme depicting the positive feedback loop between ErbB receptors, PYK2 and IL8. EGF/HRG binding to ErbB receptors activates PYK2 (pY402), which in turn, enhances STAT3 and ERK1/2 phosphorylation. Phosphorylated STAT3 enhances IL8 transcription and together with phospho-ERK1/2 potentiates MMP9 transcription. Increased levels of MMP9 facilitate cell invasion, and autocrine signaling of IL8 via its CXCR1/2 receptor prolongs ErbB-mediated signaling thereby potentiating the invasive response.

Stimulation of SKBR3 cells with IL8 induced the phosphorylation of PYK2 and activation of the STAT3, AKT and ERK1/2 pathways (Figure 6B). Similar albeit weaker effects were obtained when the low EGFR/HER2 expressing MCF7 and T47D cells were treated with IL8 (Supplementary Figure 8). Most importantly, stimulation of SKBR3 cells with EGF enhanced the transcription of IL8, mainly through activation of STAT3 (Figure 6A). PYK2 depletion inhibits EGF-induced IL8 transcription in SKBR3 cells concomitant with inhibition of STAT3 phosphorylation (Figures 4, 6A). These results suggest that PYK2-induced STAT3 phosphorylation is crucial for IL8 secretion, while IL8 is crucial for EGF-induced MMP9 transcription (Figure 7C) and for SKBR3 invasion (Figure 7B). In addition, EGF-induced IL8 secretion markedly affected the long-term activation of PYK2, STAT3 and ERK1/2 (Figure 7D), suggesting that IL8 prolongs or reinforces EGF/HRG-mediated signals and thus their cellular response. Collectively, these observations suggest a positive feedback loop that involves IL8, PYK2 and ErbB receptors (Figure 8). The model shown in Figure 8 demonstrates the role of PYK2, a common downstream effector of EGFR and IL8 receptors signaling, and its influence on both MMP9 and IL8 transcription. This model is consistent with the trans-activation of ErbB receptors by IL8 (Figure 7A) [11, 49], and with the high correlation between IL8 production in breast carcinoma and their metastatic potential (Figure 6A, Supplementary Figure 7) [12].

Our finding that PYK2 acts at the crossroads of ErbB- and IL8-receptor signaling pathways (Figure 8) to potentiate breast cancer invasion suggests that it could be an effective therapeutic target for invasive and/or metastatic breast carcinoma. Targeting of ErbB receptors is commonly used in clinic to treat breast cancer patients, in particular those with HER2 overexpression or amplification [56]. However, targeting a single receptor using monotherapy (inhibitory antibody or small molecule inhibitors) is usually not effective due to compensatory pathways and/or acquisition of resistance mechanisms. Hence, combination therapies using two or more drugs are commonly used. For example, combination of HER2 inhibitors with chemotherapy is often used to treat HER2-positive breast cancer patients [57]. Previous studies suggest that combination of anti-EGFR and anti-IL8 inhibitors markedly enhanced the anti-metastatic effect of breast cancer cells as compared to anti-EGFR alone [58], thus suggesting that combining anti-IL8 and anti-EGFR inhibitors could be an effective treatment for metastatic breast carcinoma. In light of our findings, we propose that inhibition of PYK2, a common downstream effector of both IL8 and ErbB receptors, could potentiate the impact of ErbB and/or IL8 receptor inhibitors and attenuate breast cancer invasion and metastasis.

## MATERIALS AND METHODS

### Cell culture

MCF7, T47D and SKBR3 cells were grown in RPMI (Gibco BRL, Grand Island, NY) medium supplemented with 10% fetal calf serum and a penicillin-streptomycin mixture (0.1 mg/ml; Beit Haemek, Israel).

### Antibodies and reagents

Heregulin (HRG) was purchased from PeproTech (Israel). EGF, SB225002, Hoechst 33342, TRITC-phalloidin and other chemicals were purchased from Sigma-Aldrich. Antibodies against ERK1/2, phosphorylated ERK1/2, phospho-PYK2 (Y402), STAT3 and AKT were purchased from Santa Cruz Biotechnology (Santa Cruz, CA). Antibodies against phospho-AKT (S473) and phospho-STAT3 (Y705) were purchased from Cell Signaling Technologies (Beverly, MA). Antibodies against p38 MAPK, phospho-p38MAPK and α-tubulin were purchased from Sigma. Monoclonal antibody against Paxillin was purchased from BD (San Jose, California). Polyclonal anti-PYK2 antibody was prepared as described previously [27]. Alexa-Fluor-488 donkey anti-mouse as well as anti-rabbit immunoglobulin Gs (IgGs) were purchased from Invitrogen (Carlsbad, CA). Cyanine (Cy)3-conjugated goat anti-rabbit and goat anti-mouse IgGs were purchased from Jackson ImmunoResearch Laboratories (West Grove, PA).

### DNA constructs and lentivirus production and infection

Two different shRNA sequences were used to downregulate PYK2 expression. The first one (No.1) was purchased from Sigma (TRCN00000231519), whereas the second one (No. 2) was described previously [50]. Lentivirus production and infection were conducted essentially as previously described [59]. The PYK2 shRNAs were cloned into the pLKO.1-puro lentiviral vector. Infected MCF7, SKBR3 or T47D cells were grown in selection medium containing 1 μg/ml puromycin for 72 hr.

### Immunofluorescence and confocal microscopy

Cells were grown on coverslips, washed with PBS and fixed in 4% paraformaldehyde (PFA) in PBS for 15 min at room temperature. The fixed cells were then incubated for 15 min in PBS containing 0.1 M glycine, incubated in blocking buffer containing 0.1% Triton X-100, 10% goat serum and 2% BSA in TBS for 30 min, followed by 1 hr incubation with the primary antibody, and then 1 hr incubation with the secondary antibody. After washing with PBS, the cells were incubated for 5 min with 2 μg/ml Hoechst 33342 and mounted on microscopic slides using mounting media (10 mM phosphate buffer, pH 8.0, 16.6% w/v Mowiol4–88 and 33% glycerol). The specimens were analyzed by using a confocal laser-scanning microscope (Zeiss 510; Carl Zeiss, Jena, Germany).

### RNA extraction, RT-PCR and real-time PCR analysis

RNA was purified using Tri Reagent (Sigma). cDNA was generated using oligo(dT) primer and M-MLV reverse transcriptase (Promega, Madison, WI, USA). Real-time PCR analysis was performed using SYBR Green I as a fluorescent dye, according to the manufacturer's guidelines using the ABI StepOnePlus 7500 Real-time PCR system (Applied Biosystems; Invitrogen). All experiments were carried out in triplicates and normalized to GAPDH RNA levels. Real-time PCR primers were designed using the Primerexpress software of Applied Biosystems (Invitrogen).

### Gelatin zymography

To detect MMP2 and MMP9 activity, conditioned medium was separated electrophoretically on 10% polyacrylamide/0.1% gelatin-embedded gels. The gels were then washed in 2.5% Triton X-100, and incubated at 37°C for 24 hours in 50 mM Tris-HCl (pH 7.5), containing 0.2 M NaCl, 5 mM CaCl2, 0.02% Brij 35, and stained using Coomassie Brilliant Blue (Pierce).

### Transwell migration and invasion

Cells (10^5^ cells/insert) were plated in the upper compartment of a Transwell tray (BD Bioscience, San Jose, California). The lower compartment was coated with 25 ug/ml collagen for 2 h prior to cell plating. Cells were allowed to migrate through the intervening nitrocellulose membrane for 20 hr in the presence of growth factor in the lower chamber. Thereafter, cells were fixed in 3% PFA in PBS, permeabilized in Triton X-100 (0.05%) and stained with Methyl Violet (0.02%). Non-migrating cells, growing on the upper side of the filter, were removed with a cotton swab and cells that had migrated photographed. For cell invasion assays, 10^5^ cells/insert were plated in the upper compartment of BioCoat Matrigel Chambers (BD Bioscience, San Jose, California), were allowed to invade through matrigel for 48 hr and processed as above.

## SUPPLEMENTARY FIGURES


