# Supplementary material for: PYK2 integrates growth factor and cytokine receptors signaling and potentiates breast cancer invasion via a positive feedback loop
Source: Oncotarget. 2015 Jun 10;6(26):22214–26. doi: 10.18632/oncotarget.4257 (PMC4673158; doi:10.18632/oncotarget.4257)
Supplement: Supplementary file 1 [file oncotarget-06-22214-s001.pdf]

SUPPLEMENTARY FIGURES

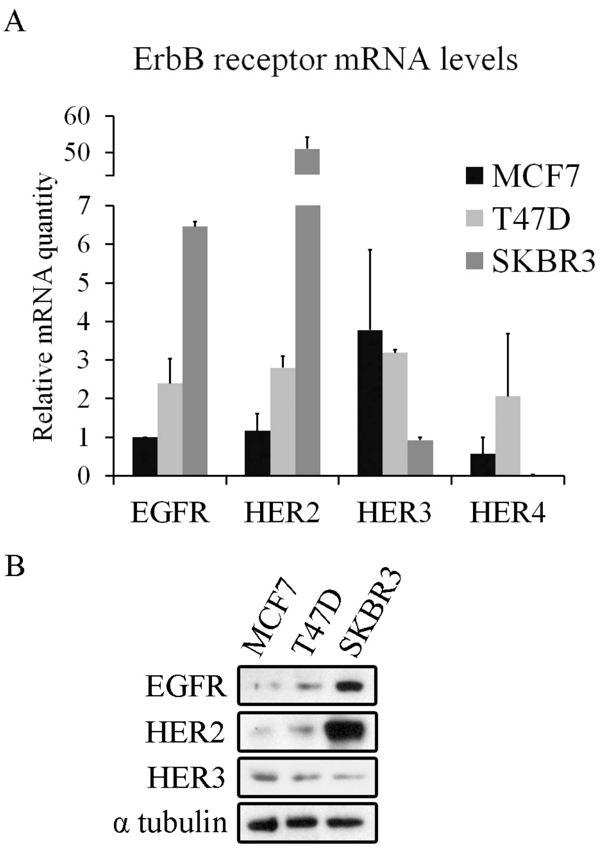

Supplementary Figure S1: Expression levels of ErbB1–4 in in MCF7, T47D and SKBR3 cells. A. The mRNA levels of ErbB1 (EGFR) and ErbB2–4 (HER2–4) in MCF7, T47D and SKBR3 cells were assessed by real-time PCR. Reproducible results were obtained in three independent experiments. The mRNA levels are presented relative to the level of EGFR in MCF7 cells (1). The mean values of three independent experiments + S.D. are shown. B. Protein levels of EGFR, HER2 and HER3 in MCF7, T47D and SKBR3 cells as assessed by Western blotting.

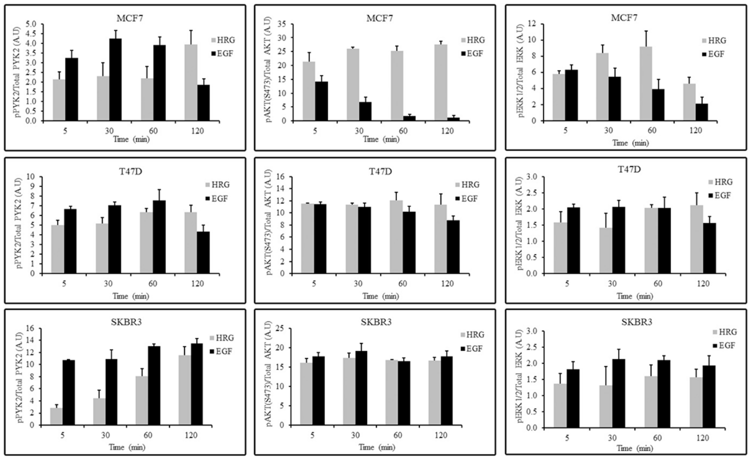

Supplementary Figure S2: Quantitative analysis of activated pathways in HRG/EGF-stimulated MCF7, T47D or SKBR3 cells. The intensity of the indicated phospho-protein and protein bands shown in Figure 1A was quantified by densitometry analysis (Image J). The results are the mean values + S.D. of three independent experiments.

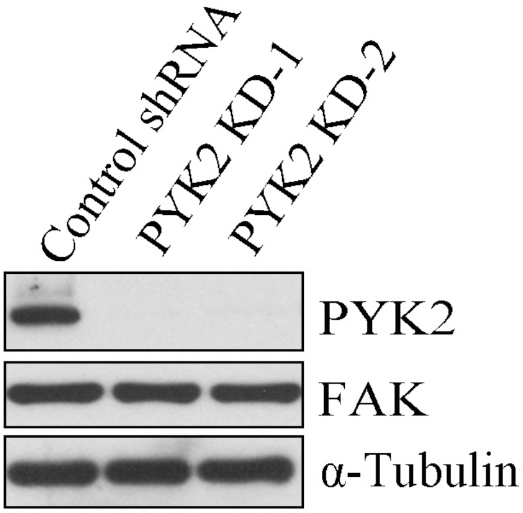

Supplementary Figure S3: Knockdown of PYK2 expression by shRNAs. SKBR3 cells were infected with lentiviruses encoding two different shRNAs (#1 and #2) against PYK2. Knockdown efficiency and specificity was assessed by WB utilizing anti-PYK2 and anti-FAK antibodies.

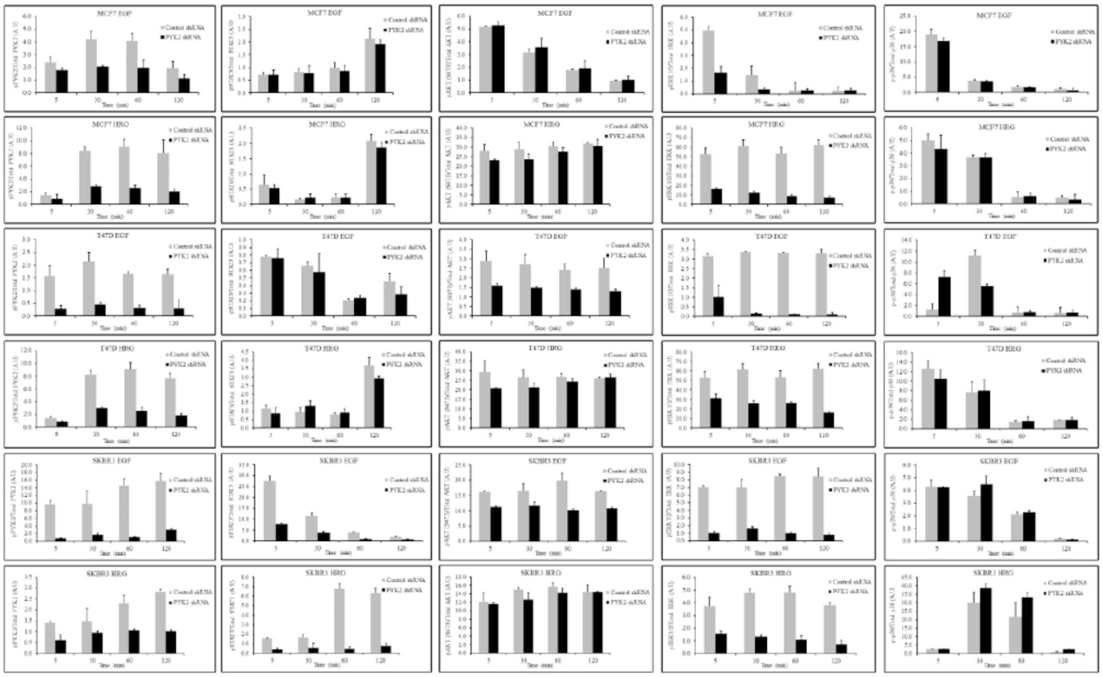

Supplementary Figure S4: Quantitative analysis of activated pathways in HRG/EGF-stimulated control and PYK2 KD MCF7, T47D or SKBR3 cells. The intensity of the indicated phospho-protein and protein bands shown in Figure 4 was quantified by densitometry analysis (Image J). The results are the mean values + S.D. of three independent experiments.

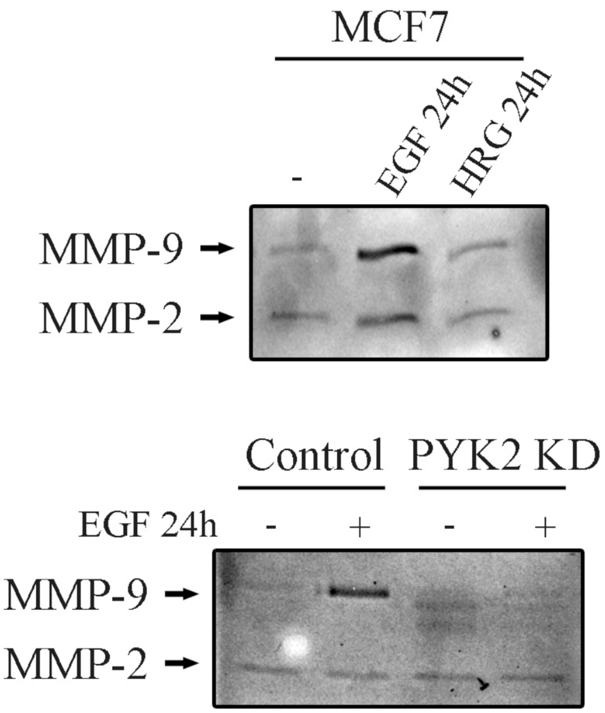

Supplementary Figure S5: PYK2 depletion inhibits MMP9 secretion in MCF7 cells. A. The activity of MMP2 and MMP9 in control and PYK2-depleted MCF7 cells in response to EGF (50 ng/ml) or HRG (10 ng/ml) was examined by gelatin zymography assay. The activity was assessed in the conditioned cell medium after 24 hrs of incubation in serum-free medium. B. The activity of MMP2 and MMP9 in control and PYK2-depleted MCF7 cells in response to EGF (50 ng/ml) or HRG (10 ng/ml) was examined by gelatin zymography assay. The activity was assessed in the conditioned cell medium after 24 hrs of incubation in serum-free medium.

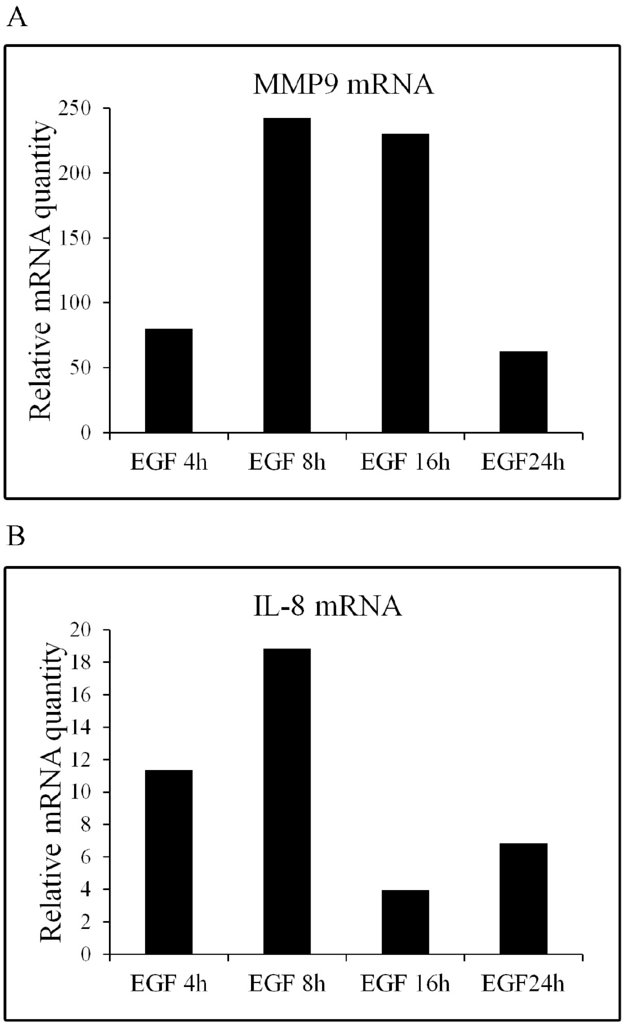

Supplementary Figure S6: MMP9 and IL-8 mRNA levels in EGF-stimulated SKBR3 cells. The mRNA levels of MMP9 **A.** and IL-8 **B.** genes in SKBR3 cells in response to various time points of EGF (50 ng/ml) were assessed by real-time PCR. Reproducible results were obtained in three independent experiments. The mRNA levels are relative to non-stimulated control cells. The mean values of three experiments are shown.

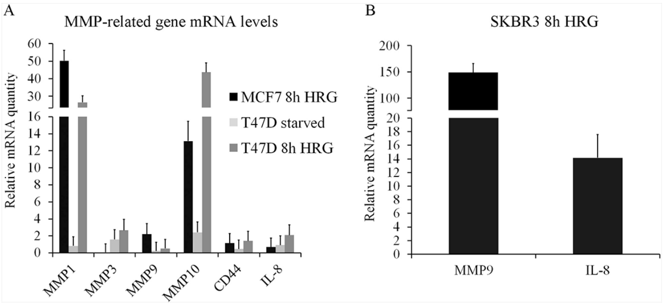

Supplementary Figure S7: Expression of MMPs and MMPs-related genes in HRG-stimulated MCF7, T47D and SKBR3 cells. The mRNA levels of the indicated MMPs and MMP-related genes in MCF7, T47D **(A)** and SKBR3 **(B)** cells in response to HRG (10 ng/ml for 8 hr) were assessed by real-time PCR. Reproducible results were obtained in three independent experiments. The mRNA levels are presented relative to non-stimulated MCF7 **A.** and SKBR3 **B.** cells. The mean values + S.D of three experiments are shown.

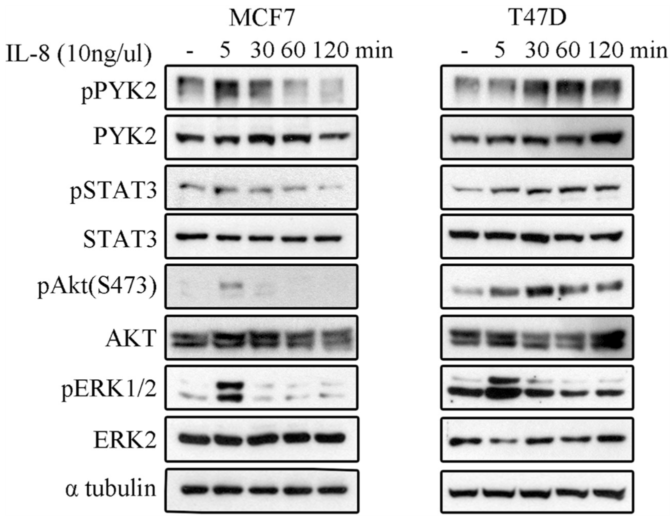

Supplementary Figure S8: S7IL-8 activates multiple signaling pathways in MCF7 and T47D cells. IL8 induces phosphorylation of PYK2 and activates the indicated downstream pathways. MCF7 and T47D cells were serum-starved for 24 hr and then stimulated with IL8 (10 ng/ml) for the indicated time periods. Total cell lysates were prepared and analyzed for the activation of different signaling pathways using WB analysis and phospho-specific antibodies as indicated. Shown are representative results of two experiments.
